# Differential miRNA-Expression as an Adjunctive Diagnostic Tool in Neuroendocrine Tumors of the Lung

**DOI:** 10.3390/cancers8040038

**Published:** 2016-03-25

**Authors:** Melanie Demes, Christoph Aszyk, Holger Bartsch, Joachim Schirren, Annette Fisseler-Eckhoff

**Affiliations:** 1 Group Practice of Pathology, 65199 Wiesbaden, Germany; demes@pathologie-wiesbaden.de; 2 Department of Pathology und Cytology, Dr. Horst-Schmidt-Hospital (HSK), 65199 Wiesbaden, Germany; c.aszyk@gmail.com (C.A.); bartsch@pathologie-wiesbaden.de (H.B.); 3 Department of Thoracic Surgery, Dr. Horst-Schmidt-Hospital (HSK), 65199 Wiesbaden, Germany; schirren@helios-kliniken.de

**Keywords:** carcinoids, LCNEC, SCLC, miRNAs, Q-PCR

## Abstract

Pulmonary malignancies with neuroendocrine differentiation represent a rare subclass of lung carcinomas, which vary in the extent of differentiation and grade of biological aggressiveness. In particular, neuroendocrine tumors are classified into well differentiated typical and atypical carcinoids as well as poorly differentiated large cell neuroendocrine and small cell lung carcinomas. Tiny MicroRNAs have been identified as reliable classifiers in distinct cancer types and seem to play important roles in cellular processes like regulation of cell growth, differentiation and apoptosis. In the present study, two different microRNAs (*miR-21* and *miR-34a*) were explored for their involvements in pathogenesis of subtypes and finally in differential diagnosis of pulmonary neuroendocrine tumors. *miR-21* was upregulated in poorly differentiated neuroendocrine tumors (mean rank: 26.8; 28.75) as compared to carcinoids (mean rank: 12.33; 12.07) with a significance of 0.00033. High-expression levels of *miR-34a* were associated with atypical carcinoids (*p* = 0.010). A close association is implicated between the elevated *miR-21* values in high-grade and *miR-34a* patterns in low-grade atypical neuroendocrine lung carcinomas, which could potentially be exploited as practical supportive markers for differential lung cancer diagnosis in routine. However, some additional extended research and validation studies are needed to utilize them as routine markers or potential molecular targets for personalized medicine.

## 1. Introduction

Generally, lung cancer is still the leading cause of cancer-related death worldwide, often diagnosed at advanced stages and with one of the poorest prognoses of all types of cancer. Within the last decades, a linearly increasing incidence of neuroendocrine tumors is noticeable [1].

Neuroendocrine tumors (NETs) are generally found throughout the human body, but most commonly in the small intestine (30.4%) and the lung (29.8%) [2,3]. Lung tumors with neuroendocrine differentiation are further subdivided into four subtypes, low grade (G1) typical carcinoid (TC), intermediate grade (G2) atypical carcinoid (AC), high grade (G3) large cell neuroendocrine carcinoma (LCNEC) and small cell lung carcinoma (SCLC). When looking at the distribution of NET subtypes, carcinoids comprise for 1% to 2%, LCNECs for 3% and SCLCs for 15% to 20% of all lung cancer malignancies [4]. LCNECs and SCLCs are associated with older age and more smoking compared to TCs and ACs. Generally, carcinoid tumors (especially those located in the periphery) are accompanied by milder symptoms and are occasionally diagnosed by chance in an asymptomatic patient [5]. Besides epidemiologic differences, there are also some clinical and genetic variations between low grade carcinoid tumors and high grade SCLCs and LCNECs, although they share a similar neuroendocrine phenotype. Neuroendocrine tumors may differ in the proportion of necrosis, mitotic activity, palisading structure, trabecular pattern and organoid nesting [3]. However, the classification of NE lung tumors can still be problematic (mainly in small biopsies) due to their similar morphological patterns which has an amazing influence on therapy and prognostic outcome.

Low grade TC and AC patients generally undergo surgical treatment with good disease-free survival rates, while high grade LCNEC and SCLC patients are treated with chemotherapeutic drugs with poor disease-free survival rates. Thus, one of the greatest challenges in the field of cancer research is the identification of reliable biomarkers for differential diagnosis and consequently to ensure an accurate prognosis as well as faithful therapy prediction.

For this purpose, microRibonucleic acids (miRNAs) have gained a lot of interest regarding their use as potential differential diagnostic markers and as a hopeful tool for the development of novel therapeutic approaches [4,5]. It is well known that those small non-coding RNA molecules (~20 to 25 nucleotides) play important roles in the pathogenesis of various diseases, including cancer. They are able to regulate many cellular processes (on posttranscriptional level) like proliferation, differentiation, angiogenesis, metabolism and apoptosis and seem to play a complex role in lung cancer [6,7]. miRNAs that are involved in cancer are also known as oncomirs, and *miR-21* is one of the first being categorized as such [8]. A number of targets for *miR-21* have been experimentally identified and most of them are tumor suppressors. Some prominent examples include *PTEN*, *Tropomyosin*, *Bcl2*, *JAG1* and *TGFBRII* [9,10,11,12,13]. Additional to the above-mentioned references, Lee *et al.* describes a differential expression of *miR-21* according to the histological subtype of pulmonary neuroendocrine tumors as an interesting novel molecular tool [14].

It has been described that *miR-34a* targets the *SIRT1* gene together with *p53*, which results in a suppression of apoptosis through the *SIRT1-p53* pathway [15]. As novel molecular biomarkers and potential therapeutic agents (in contrast to chemotherapeutic drugs), those extracellular miRNAs have the advantage of being minimally invasive, highly sensitive and specific markers in the diagnosis of cancer.

For miRNA study, we filtered the number of significant miRNAs which were differently expressed in primary pulmonary tumors. Therefore, after literature research, we decided to study the role of *miR-21* and *-34* in tumorgenesis of neuroendocrine tumors with respect to the histological subtype.

## 2. Results

### 2.1. Baseline Characteristics and Pathologic Assessment

This retrospective study assessed the miRNA expression status of 38 neuroendocrine pulmonary tumors of 17 (44.7%) males and 21 (55.3%) females with a median age of 66. The study includes surgical resection tissues and biopsies. The distribution of the age is skewed to the left with a minimum age of 42 years and a maximum age of 82 years.

Further pseudonymous patients and tumor characteristics such as the cancer type and its tumor or nodal descriptors are summarized in Table 1.

**Figure 1:**
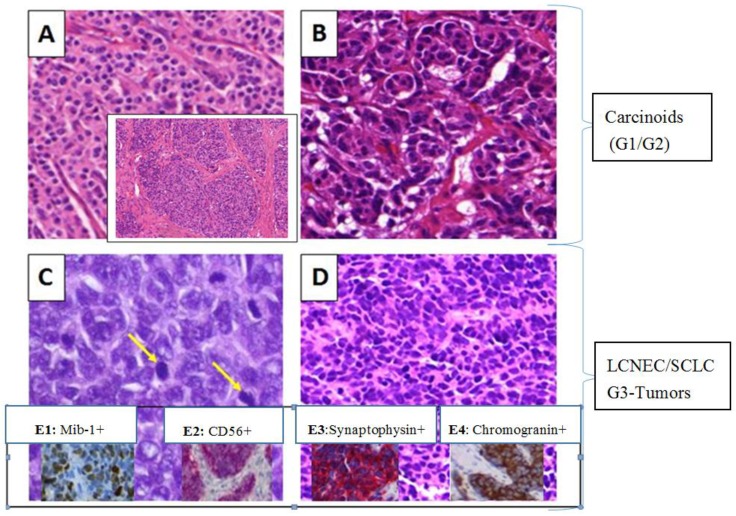
H&E (**A**–**D**) as well as IHC (**E1**–**E4**) stained specimens of four different NET subtypes of the lung. Typical carcinoid (**A**); atypical carcinoid (**B**); large cell neuroendocrine carcinoma (**C**); and small cell lung carcinoma (**D**) are shown (MiraxDesk scanned). Yellow arrows indicate high mitotic rates. (**E1**–**E4**) IHC markers: (**E1**) LCNEC, MIB-1 analysis (tumor cell proliferation rate); (**E2**) LCNEC, positive for CD56; (**E3**) SCLC, positive for synaptophsin; and (**E4**) LCNEC, positive for chromogranin (MiraxDesk scanned).

All carcinomas were histologically confirmed neuroendocrine tumors of the lung (Figure 1), six (15.8%) patients were diagnosed for atypical carcinoids, 14 (36.8%) for typical carcinoids, 10 (26.3%) for large cell neuroendocrine carcinomas and eight (21.1%) for small cell lung carcinomas (Table 1).

- 20 carcinoids (G1/G2), 52.6%
- 18 LCNECs/SCLCs (G3), 47.4%

As visible in Figure 1A,B, carcinoids had a typical characteristic hematoxylin and eosin (H&E) stained histological pattern of organoid, trabecular, palisading and spindle-like cells.

TCs showed no necrosis, had a tumor size equal or less than 0.5 cm and a mitotic rate less than 2 per 10 high power fields (HPFs) (Figure 1A). ACs (Figure 1B) comprised small punctated necrosis or areas of coagulative necrosis and a mitoses number between 2 to 10 per 10 HPFs. ACs were sometimes difficult to differentiate from TCs. The appearance of LCNECs (Figure 1C) was often accompanied by large tumor cells with low nuclear-to-cytoplasmic ratio and frequently detectable nucleoli. Moreover, necrosis was typically present in large zones with a high mitotic rate (11 or greater per 10 HPFs), see yellow arrows in Figure 1C. SCLCs (Figure 1D) were generally small in size with scant cytoplasm. They showed high mitotic rates combined with frequent large areas of necrosis. In order to underline the differential diagnosis of tumor typing, immunohistochemical (IHC) assessments were taken into account. As illustrated in Figure 1 (E1–E4), typical immunohistochemical stainings with a panel of four conventional markers such as CD56 (Figure 1E2), synaptophysin (Figure 1E3) and chromogranin (Figure 1E4) were performed on available tissue to confirm the neuroendocrine phenotype. In addition, the tumor cell proliferation rate (or mitotic rate) which differs between low-grade or high-grade NEs was visualized through the MIB-1 antibody (Figure 1E1) [3].

### 2.2. Differential miRNA Expression in Pulmonary Subtypes of Neuroendocrine Tumors

**Figure 2:**
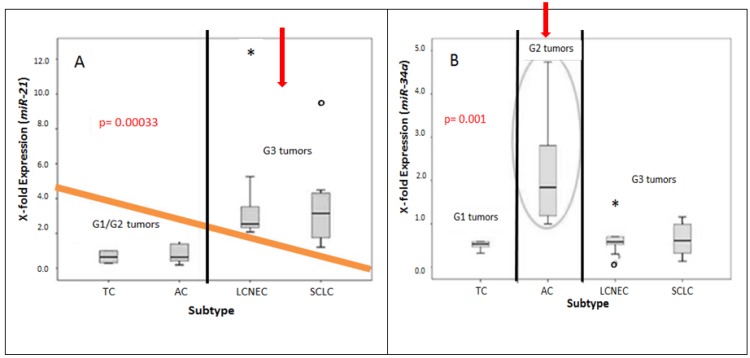
Comparative gene expression analysis of *miR-34a* and *miR-21* in pulmonary neuroendocrine subtypes (*n* = 38). Calculated X-fold expression (2^−ΔΔ*C*t^ – y-axis) is plotted against all four distinct subtypes (x-axis). *miR-21* is differentially expressed in carcinoids and high-grade neuroendocrine lung tumors (**A**). Atypical carcinoids (G2) show a higher *miR-34a* expression level as compared to G1 and G3 neuroendocrine tumors (**B**).

Figure 2 illustrates the correlation between the expression pattern of miRNAs and neuroendocrine subtypes.

TCs (Average: 0.833) and ACs (Average: 0.846) showed a much lower x-fold expression of *miR-21* as compared to LCNECs (Averageg: 3.493) and SCLCs (Average: 3.633).

For TCs and ACs, the highest *miR-21* expression reached a value of 1.392 and 2.180, respectively. Contrarily, LCNECs and SCLCs show high *miR-21* expression values with a maximum of 12.346 and 9.448. Thus, *miR-21* seems to be higher expressed in LCNECs and SCLCs (G3 tumors) as compared to carcinoids (G1/G2).

The mean ranks of *miR-21* differed between low-grade and high-grade tumors with an asymptotic significance of 0.00033. The “ONEWAY” ANOVA analysis indicated a significance of 0.006.

According to the “Bonferroni”-correction (α_i_ = 0.05/amount of tests (n)), the null hypothesis (H_0_) was rejected. Thus, *miR-21* seems to be differentially expressed in low grade and high grade tumors. The mean ranks of *miR-34a* differed between typical and atypical carcinoids with an asymptotic significance of 0.010 underlined by the ONEWAY ANOVA analysis (*p* = 0.015), too.

**Figure 3:**
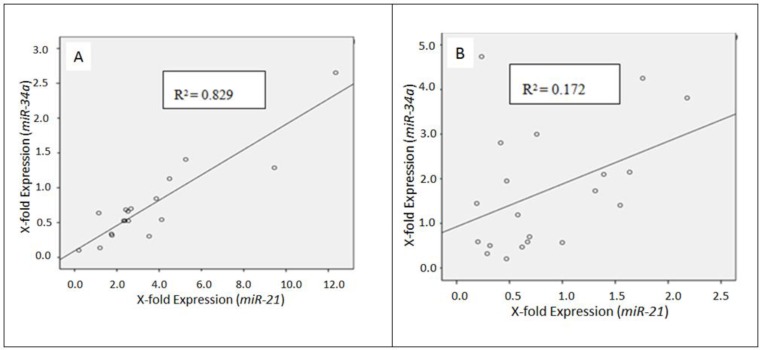
Relationship between *miR-34a* and *miR-21* in carcinoids (**A**) and G3-neuroendocrine tumors (**B**), respectively. The drawn “Line of Best Fit” as well as the coefficient of determination (R2) describes the association of the two variables.

The relationship between the variables of interests, *miR-34a* and *miR-21*, in low-grade (G1–G2) and high-grade (G3) neuroendocrine tumors was further analyzed by a scatterplot and resulting correlation value (Figure 3).

The correlation value of carcinoid tumors was 0.829. This match was judged to be rather good, presenting a positive correlation between *miR-34a* and *miR-21* in carcinoid tumors. This result could be explained by a higher number of typical carcinoids as compared to atypical ones.

The match of *miR-34a* and *miR-21* in high-grade (G3) tumors was rather poor, indicated by a correlation value of 0.172 (Figure 3).

## 3. Discussion

Consistent with reference data from the literature, lung cancer in general develops and is diagnosed at advanced ages (median age 66 years) due to lifestyle habits (e.g., smoking) and environmental conditions (e.g., particulate air pollution). Thus, almost all patients diagnosed for LCNEC or SCLC are cigarette smokers; 60% to 80% of TC/AC patients are also associated with smoking and significantly younger age [16,17,18,19,20].

Neuroendocrine tumors of the lung comprise for approximately 20% to 25% of all invasive lung malignancies [21]. Looking at the particular distribution of investigated NET subtypes, TCs and LCNECs are the most abundant, followed by SCLCs and ACs.

According to the literature, carcinoids in general comprise 1% to 2% of all lung cancers, from which 10% accounts for ACs. In contrast, LCNECs are involved in 3% and SCLCs in 15 to 20% of all lung carcinoma cases [22]. This distribution is not reflected in the investigated study, due to the strong pre-selection of suitable cases to achieve a homologous collective with an appropriate tissue quality for reliable Quantitative Polymerase- Chain-Reaction analysis [23,24,25,26,27,28].

The discrimination between distinct neuroendocrine tumors of the lung which is based on histological aspects is a challenge for pathologists. We focused on miRNAs as a supporting tool for differentiation. In our study miRNAs were found to be robust presenting reproducible results. This is very important for routine diagnostic purposes. The increasing expression level of *miR-21* was correlated to an increasing degree of malignancy of neuroendocrine pulmonary tumors. Thus, *miR-21* is a potential diagnostic marker whose expression differs between high-grade and low-grade neuroendocrine tumors. Other studies show also a significant up-regulation of *miR-21* in patients with high grade NETs (LCNEC and SCLC) [29,30,31]. Mairinger and colleagues found a differently expression pattern of miRNAs in pulmonary neuroendocrine tumors (*n* = 12), too [32]. Another study investigates the expression pattern of three selected miRNAs (*miR-21*, *miR-155* and *let-7a*) of 63 surgically resected pulmonary neuroendocrine tumors. The expression level of *miR-21* and *miR-155* was significant higher in high-grade NE carcinomas than in carcinoid tumors (each *p* < 0.001) [33]. *miR-21* has also been associated with a wide variety of other cancers (e.g., breast, ovaries, colon and prostate) [30,31,32,33,34]. Currently, a research group has shown an increase in apoptosis in DU145 and PC-3 cells after blocking *miR-21* function in glioblastoma cell lines [34]. Thus, *miR-21* directs cell growth by inhibiting apoptosis [35,36,37,38,39,40].

Lee and colleagues demonstrate no significant differences in the miR-34 expression pattern between the four described neuroendocrine subtypes [14]. Contrarily, our study showed an elevated *miR-34a* expression level in typical carcinoids. The utilization of *miR-34a* as differential marker would be a great advantage due to high morphological similarities between typical and atypical carcinoids.

According to the literature, *miR-34a* may inhibit SIRT1 which leads to an increased level of acetylated p53 and expression of p21 and PUMA regulating the cell cycle and apoptosis, respectively [41]. Thus, *miR-34a* functions as a tumor suppressor and could be a potential therapeutic target of interest.

## 4. Materials and Methods

### 4.1. Patients and Tissue Samples

Archival formalin fixed paraffin embedded (FFPE) human neuroendocrine lung cancer samples of 38 pseudonymous patients were investigated.

Neuroendocrine features were additional characterized by auxiliary different diagnostic immunohistochemical (IHC) markers (*i.e.*, chromogranin, synaptophysin and CD56).

For analytical processes, the scraped tissue was characterized with respect to inflammatory cells as well as the proportion of necrosis and viable tumor cells.

### 4.2. RNA Isolation

RNA was prepared from 12–20 serial 5 µm-thick and H&E) stained paraffin sections. The mircodissected material was manually isolated from deparaffinizated samples according to the instructions provided by the manufacturer (RecoverAll^TM^ Total Nucleic Acid Isolation Kit, Ambion). Protein digestion was carried out over night with 4 µL protease K. Additionally, optical density (OD_260/280_) measurements and agarose gel electrophoresis were used to estimate the quality of isolated RNA and amplified products, respectively.

### 4.3. Primer Sequences

The utilized stem-loop primers were specific for the sequence of interest. The illustrated sequences are shown without stem-loop structure in Table 2 [42].

### 4.4. Quantitative Analysis of miRNAs in Neuroendocrine Tumors of the Lung

Relative comparative analysis (2^−ΔΔCT^-method) of miRNA expression in normal and tumor tissue of the lung was carried out in a two-step mechanism. As a first step, isolated miRNA was reverse transcribed into its single-stranded complementary (c)DNA using TaqMan^TM^ MicroRNA Reverse Transcription Kit (Applied Biosystems, Carlsbad, CA, USA) [42]. In the second step, the cDNA was amplified and simultaneously quantified by Q-PCR.

The efficiency of Q-PCR was determined via calibration dilution curve and slope calculation using a conventional Human Reference RNA (Agilent Technologies, Santa Clara, CA, USA). As endogenous control, several housekeeping markers have been tested with TaqMan^TM^ MicroRNA Assay U47 (Applied Biosystems) being the most suitable one. In addition, the Q-PCR was performed as duplicate for each test sample. The x-fold miRNA expression of the respective tumor was determined through the application of the 2^−^^ΔΔCT^-formula with individual normal lung tissue as calibrator.

### 4.5. Statistics and Mathematics

The SPSS 17.0 (IBM, Ehningen, Germany) were used for statistical analysis. Differential expression of miRNA in histological subtypes was characterized using “ONEWAY” ANOVA analysis, Kruskal-Wallis, Mann-Whitney-U and Wilcoxon-W tests. Histograms, boxplots and scatterplots also visualize and describe the association of variables and histological subtypes, respectively. Correction for the significance-value p was applied by using the Bonferroni (α_i_) correction (α_i_ = 0.05/amount of tests).

## 5. Conclusions

Looking at the current clinical practice, one of the greatest challenges with pulmonary NE tumors is that there is no optimal therapy established and the tumors are mostly diagnosed at late-stage with poor prognosis. Moreover, a clear differentiation between individual subtypes is sometimes a challenge. Hence, there is a great need in developing faithful biomarkers for differential diagnosis which has a great influence on prognosis and treatment options. Several independent studies illustrate important roles of miRNAs in tumorgenesis and they mention a lot of different oncologic targets (e.g., silencing effects on pro-apoptotic factors).

Summarizing the outcome of this study, miRNAs seem to be differentially expressed in neuroendocrine tumors of the lung. A close association was implicated between the elevated *miR-21* in high-grade and *miR-34a* in low grade atypical neuroendocrine lung carcinomas, which could potentially be exploited as practical supportive markers for differential lung cancer diagnosis in routine. However, some additional research and validation studies are needed to utilize them as routine markers or potential molecular targets for personalized medicine.

Expansion of the current collection is planned. In addition to the differently diagnostic role of miRNAs, the predictive and prognostic value in neuroendocrine tumors will be investigated. It would also be interesting to assess miRNA levels in neuroendocrine tumors of the lung compared to those in non-small cell lung carcinomas as well as mesenchymal tumors, paragangliomas and intrapulmonary metastases of well-differentiated breast or prostate carcinomas for differential purposes. Another starting point for novel targeted therapies is for example functional analytics on the basis of proteins or messenger RNAs to find new therapeutic targets or unknown resistances against chemotherapeutic drugs (especially in neuroendocrine tumors).

## Figures and Tables

**Table 1:** Summary of pseudonymous cases with respect to histological characteristics.

Characteristic		Absolute Number	Percentage (%)
Cases		*38*	*100*
CANCER SUBTYPE ^1^	*AC*	*6*	*15.8*
	*TC*	*14*	*36.8*
	*LCNEC*	*10*	*26.3*
	*SCLC*	*8*	*21.1*
LOCATION ^2^	*ML*	*5*	*13.2*
	*LU*	*13*	*34.2*
	*RU*	*4*	*10.5*
	*LL*	*8*	*21.1*
	*RL*	*8*	*21.1*
HISTOLOGICAL GRADING (G)	*G1*	*14*	*36.8*
	*G2*	*6*	*15.8*
	*G3*	*18*	*47.4*
TUMOR (T) STAGE	*T1a*	*15*	*39.5*
	*T1b*	*7*	*18.4*
	*T2a*	*7*	*18.4*
	*T2b*	*1*	*2.6*
	*pT3*	*7*	*18.4*
	*n.a.* ^3^	*1*	*2.6*
NODAL (N) STAGE	*N0*	*27*	*71.1*
	*N1*	*5*	*13.2*
	*N2*	*3*	*7.9*
	*N3*	*2*	*5.3*
	*n.a.*	*1*	*2.6*

^1^ AC = atypical carcinoid; TC = typical carcinoid; LCNEC = large cell neuroendocrine tumor; SCLC = small cell lung cancer; ^2^ ML = middle lobe; LU = left upper lobe; LL = left lower lobe; RU = right upper lobe; RL = right lower lobe; ^3^ n.a. = not available.

**Table 2:** Sequence of miRNAs studied in neuroendocrine tumors of the lung.

miRNA	Mature miRNA Sequence (5′–3′)	Length	Species	Melting Temperature
***21***	UAG CUU AUC AGA CUG AUG UUG A	22 bp	Homo sapiens	49.2 °C
***34a***	UGG CAG UGU CUU AGC UGG UUG U	22 bp	Homo sapiens	53.0 °C
***U47 (endogenous control)***	TAA TGA TTC TGC CAA ATG AAA TAT AAT GAT ATC ACT GTA AAA CCG TTC CAT TTT GAT TCT GAG GT	65 bp	Homo sapiens	67.2 °C
